# Integration of Metabolomics and Transcriptomics Reveals a Complex Diet of *Mycobacterium tuberculosis* during Early Macrophage Infection

**DOI:** 10.1128/mSystems.00057-17

**Published:** 2017-08-22

**Authors:** Michael Zimmermann, Maria Kogadeeva, Martin Gengenbacher, Gayle McEwen, Hans-Joachim Mollenkopf, Nicola Zamboni, Stefan Hugo Ernst Kaufmann, Uwe Sauer

**Affiliations:** a Department of Biology, Institute of Molecular Systems Biology, ETH Zurich, Zurich, Switzerland; b Department of Immunology, Max Planck Institute for Infection Biology, Berlin, Germany; c Department of Microbiology and Immunology, Yong Loo Lin School of Medicine, National University of Singapore, Singapore, Singapore; d Core Facility Microarray/Genomics, Max Planck Institute for Infection Biology, Berlin, Germany; University of California, San Diego

**Keywords:** *Mycobacterium tuberculosis*, host-pathogen interactions, metabolism, systems biology

## Abstract

Nutrient acquisition from the host environment is crucial for the survival of intracellular pathogens, but conceptual and technical challenges limit our knowledge of pathogen diets. To overcome some of these technical roadblocks, we exploited an experimentally accessible model for early infection of human macrophages by *Mycobacterium tuberculosis*, the etiological agent of tuberculosis, to study host-pathogen interactions with a multi-omics approach. We collected metabolomics and complete transcriptome RNA sequencing (dual RNA-seq) data of the infected macrophages, integrated them in a genome-wide reaction pair network, and identified metabolic subnetworks in host cells and *M. tuberculosis* that are modularly regulated during infection. Up- and downregulation of these metabolic subnetworks suggested that the pathogen utilizes a wide range of host-derived compounds, concomitant with the measured metabolic and transcriptional changes in both bacteria and host. To quantify metabolic interactions between the host and intracellular pathogen, we used a combined genome-scale model of macrophage and *M. tuberculosis* metabolism constrained by the dual RNA-seq data. Metabolic flux balance analysis predicted coutilization of a total of 33 different carbon sources and enabled us to distinguish between the pathogen’s substrates directly used as biomass precursors and the ones further metabolized to gain energy or to synthesize building blocks. This multiple-substrate fueling confers high robustness to interventions with the pathogen’s metabolism. The presented approach combining multi-omics data as a starting point to simulate system-wide host-pathogen metabolic interactions is a useful tool to better understand the intracellular lifestyle of pathogens and their metabolic robustness and resistance to metabolic interventions.

**IMPORTANCE** The nutrients consumed by intracellular pathogens are mostly unknown. This is mainly due to the challenge of disentangling host and pathogen metabolism sharing the majority of metabolic pathways and hence metabolites. Here, we investigated the metabolic changes of *Mycobacterium tuberculosis*, the causative agent of tuberculosis, and its human host cell during early infection. To this aim, we combined gene expression data of both organisms and metabolite changes during the course of infection through integration into a genome-wide metabolic network. This led to the identification of infection-specific metabolic alterations, which we further exploited to model host-pathogen interactions quantitatively by flux balance analysis. These *in silico* data suggested that tubercle bacilli consume up to 33 different nutrients during early macrophage infection, which the bacteria utilize to generate energy and biomass to establish intracellular growth. Such multisubstrate fueling strategy renders the pathogen’s metabolism robust toward perturbations, such as innate immune responses or antibiotic treatments.

## Abstract (precis)

The nutrients consumed by intracellular pathogens are mostly unknown. This is mainly due to the challenge of disentangling host and pathogen metabolism sharing the majority of metabolic pathways and hence metabolites. Here, we investigated the metabolic changes of *Mycobacterium tuberculosis*, the causative agent of tuberculosis, and its human host cell during early infection. To this aim, we combined gene expression data of both organisms and metabolite changes during the course of infection through integration into a genome-wide metabolic network. This led to the identification of infection-specific metabolic alterations, which we further exploited to model host-pathogen interactions quantitatively by flux balance analysis. These *in silico* data suggested that tubercle bacilli consume up to 33 different nutrients during early macrophage infection, which the bacteria utilize to generate energy and biomass to establish intracellular growth. Such multisubstrate fueling strategy renders the pathogen’s metabolism robust toward perturbations, such as innate immune responses or antibiotic treatments.

## INTRODUCTION

Intracellular pathogens acquire nutrients from the host environment during infection to generate energy and biomass for survival and replication (1–3). Whether a given substrate is consumed by pathogens depends on its availability at the site of infection and the pathogen’s metabolic capacity (4, 5). Although nutrient uptake and utilization are the basis of an organism’s physiology, it is poorly understood for most intracellular pathogens during infection. Investigations of *in vivo* physiology during infection are conceptually and technically challenging, and evidence for intracellular nutrient usage is mostly indirect. The standard method to probe intracellular pathogen uptake of a specific substrate is based on infection phenotypes of auxotrophic strains (6). More direct evidence may be obtained by monitoring ^13^C incorporation into proteinogenic amino acids of bacteria infecting prelabeled host cells (7). These approaches provide insights into the consumption of host molecules as biomass precursors, but they typically fail to identify energy sources (8).

Development of omics technologies has enabled the assessment of system-wide changes of transcript and protein levels as surrogate measurements of metabolic rearrangements during infection (9–11). Conceptually, the main difficulty of omics experiments is to define appropriate reference conditions that allow distinguishing metabolic interactions from indirect effects, such as host response-triggered stresses that influence gene expression. In a recent study of *Shigella flexneri* infection, targeted metabolite measurements helped to identify a major carbon flux from the human host cell through the pathogen and back to the host (12). Although such direct analysis of metabolites would be ideal for understanding a pathogen’s *in vivo* physiology, it is hampered by the large overlap of host and pathogen pathways (13). Therefore, physiological analysis of intracellular pathogens requires a combination of complementary (omics) measurements and integration strategies for data interpretation.

*Mycobacterium tuberculosis*, the causative agent of tuberculosis, led to an estimated 10.4 million new cases and 1.8 million deaths in 2015 (14). Airborne bacilli typically infect the lungs, where the bacteria are ingested by alveolar macrophages. *M. tuberculosis* successfully evades killing by macrophages by residing in modified vacuoles for replication and prolonged survival upon initiation of the adaptive immune response (15–17). To colonize its niche, *M. tuberculosis* resists host defense while acquiring nutrients from the host environment to generate energy and biomass for its survival and replication. To identify consumed nutrients and engaged metabolic pathways during infection, growth of auxotrophic *M. tuberculosis* strains was assessed in cellular and animal infection models (6). These studies revealed crucial roles of amino acid metabolism (18–20), mycobacterial lipid uptake and breakdown (21–24), lipid-based fueling of central carbon metabolism (25, 26), and gluconeogenesis (27, 28) but intriguingly, also carbohydrate uptake and utilization (29, 30). Genome-wide transcript data of intracellular mycobacteria aimed at capturing more subtle metabolic adaptations during infection, such as regulation of generally essential genes and genes that are not causing pronounced infection phenotypes upon perturbation. Targeted inspection of transcriptional changes in metabolic pathways and ontology enrichment analysis underscored the activation of lipid-related metabolic pathways and general stress responses upon entry into the host cell (31–35). Tracking biomass incorporation by cold or hot substrate labeling strategies directly demonstrated *in vivo* utilization of both specific lipids and amino acids by *M. tuberculosis* (7, 24). However, despite remarkable progress in understanding mycobacterial metabolism of recent years, many aspects of intracellular nutrient acquisition by *M. tuberculosis* remain puzzling (36).

Inspired by the fact that extensive cometabolism of nutrients has previously been suggested for *M. tuberculosis* (7, 37), we develop here an approach to (i) identify putative substrates of the intracellular pathogen, (ii) quantify their relative utilization, (iii) assess their fate in the pathogen’s metabolism, and (iv) infer metabolic consequences in the host caused by bacterial metabolism. Early infection of human macrophage-like cells with *M. tuberculosis* served as a model system for the integration of dynamic metabolite and transcript data to infer metabolic host-pathogen interactions. We collected samples for both of these measurements from the same cultures and overlaid the resulting data onto a genome-wide metabolic reaction pair network to identify metabolic subnetworks of particular importance during infection. We then integrated the detected transcriptional changes into a combined genome-scale model of human macrophages and *M. tuberculosis* (38) to simulate the host-pathogen metabolic interactions *in silico*. Our analyses revealed the pathogen’s coutilization of up to 33 different nutrients during macrophage infection, of which we predicted 3 to be used solely as biomass precursors, whereas the rest are further metabolized for energy generation and precursor formation. On the basis of these findings, we propose that such multiple-substrate fueling confers high robustness to interventions of the pathogen’s metabolism.

## RESULTS

### Measured metabolites are mainly derived from the host.

To study metabolic host-pathogen interactions during the early stage of infection, we infected THP-1 cells with *M. tuberculosis* H37Rv after induced differentiation into macrophages (39). This system was selected for the following reasons: (i) the human origin of the host in contrast to murine systems, (ii) the highly reproducible characteristics of a stable cell line in contrast to primary cell isolates, (iii) immediate intracellular replication upon infection compared to extensive initial bacterial killing of other model systems (34, 39).

Given the high conservation of metabolic processes in biological systems, the vast majority of metabolites, such as nucleotides, amino acids, and central carbon metabolites, are commonly found across organisms. Hence, measured metabolites typically cannot be assigned to a particular organism in mixed samples, in sharp contrast to DNA, RNA, and proteins whose sequences are mostly species specific. The short biological turnover time of metabolites precludes physical separation of species prior to metabolite measurement; hence, infection samples contain both host- and pathogen-derived metabolites. Therefore, prior to setting up the infection, we performed a metabolomics spike-in experiment of different mixtures of separately cultured THP-1 macrophages and tubercle bacilli (cell-to-bacterium ratios of 1:0, 1:5, 1:10, and 1:20) to evaluate the potential contribution of either organism to each of the assessed metabolite pools.

**FIG 1:**
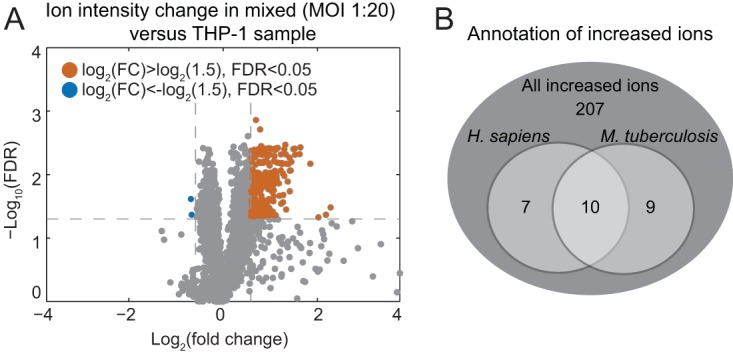
Comparison of the THP-1 metabolome with the mixed metabolome of THP-1 cells and *M. tuberculosis*. (A) Volcano plot of pairwise comparisons of all detected ions using an unpaired *t* test for the metabolome of THP-1 cells alone and mixed with bacterial cells. Data shown are measurements for three biological replicates and two technical replicates. FC, fold change; FDR, false-discovery rate. (B) Venn diagram showing the annotation of increased ions upon spiking THP-1 cells with *M. tuberculosis*. Data are shown for the maximal ratio of host cells to bacteria (1:20).

Untargeted metabolite measurements were applied for their high measurement coverage (40), which resulted in the detection of 4,593 ions in total. Pairwise comparison (*t* test) of ion intensities revealed that 233 (5%) of all detected ions showed a significant increase (fold change of >1.5; false-discovery rate [FDR] of <0.05) in the presence of the maximally assayed, nonphysiological bacterial load of 20 bacteria per single THP-1 cell compared to THP-1 alone (Fig. 1A; also see Fig. S1 in the supplemental material). Only two ions were found to have significantly decreased intensities, demonstrating that addition of bacterial biomass to the samples does not negatively interfere with the measurement, e.g., through matrix effects (41). To unravel the identity of the increased ions upon the addition of bacteria to THP-1 cells, we adapted a metabolite reference list that aggregated the Kyoto Encyclopedia of Genes and Genomes (KEGG) metabolite repository specific to *M. tuberculosis* H37Rv (mtu, 3,414 metabolites), *Homo sapiens* (hsa, 2,915 metabolites) (42), and the *M. tuberculosis* Mtb Lipid Database (http://mrl.colostate.edu/mtb/) and MycoMass (http://www.brighamandwomens.org/research/depts/medicine/rheumatology/Labs/Moody/default.aspx) databases of mycobacterial lipids (10,424 metabolites in total) (43). Out of 233 increased ions, 26 could be annotated, of which 9 were likely derived from *M. tuberculosis* due to their species specificity (Fig. 1B and Table S1). Since only a few human metabolites changed upon bacterial spiking (17 of 514 annotated metabolites), we conclude that detected metabolite signals mainly reflect host metabolites and that mycobacterial metabolites can be monitored only if they are unique and relatively abundant in the bacteria.

10.1128/mSystems.00057-17.1 FIG S1  Comparison of the THP-1 metabolome with the mixed metabolome of THP-1 cells and *M. tuberculosis* (ratio 1:20). (A) Volcano plot of pairwise comparison of all ions with KEGG *H. sapiens* (hsa) annotations using an unpaired *t* test. (B) Volcano plot of pairwise comparison of all ions with KEGG *M. tuberculosis* (mtu) annotations using an unpaired *t* test. (C) Volcano plot of pairwise comparison of all ions with lipid database annotations using an unpaired *t* test. (D) Volcano plot of the intersection of increasing ions with KEGG *H. sapiens* (hsa) and lipid database annotations. Annotations were filtered for ion adducts. All data come from at least three biological replicates and two technical replicates. FC, fold change; FDR, false-discovery rate. Download FIG S1, EPS file, 1.7 MB. Copyright © 2017 Zimmermann et al. 2017 Zimmermann et al. This content is distributed under the terms of the Creative Commons Attribution 4.0 International license.

10.1128/mSystems.00057-17.7 TABLE S1  Metabolomics data of irradiated tubercle bacillus spiking experiment and THP-1 infection time course experiment. Pathway enrichment analysis results of metabolomics infection time course data. Download TABLE S1, XLSX file, 3.6 MB. Copyright © 2017 Zimmermann et al. 2017 Zimmermann et al. This content is distributed under the terms of the Creative Commons Attribution 4.0 International license.

### Dynamic metabolite changes during infection.

**FIG 2:**
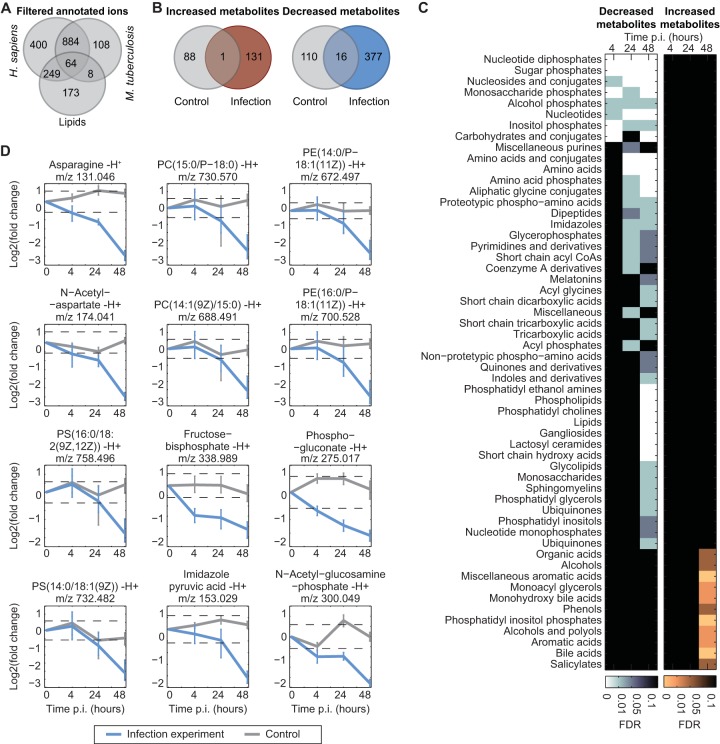
Analysis of changes in the host metabolome during *M. tuberculosis* infection. (A) Annotation statistics of all detected ions after the filtering procedure. (B) Comparison of the number of changing metabolites in control and infection samples at 48 h p.i. defined using a fold change (FC) cutoff of |log_2_ FC| > log_2_ 1.5 and FDR < 0.05. (C) Chemical class enrichment analysis of changing metabolites at each time point. Metabolites were displayed only if the enrichment false-discovery rate (FDR) was <0.1. (D) Temporal profiles of metabolites representative of certain metabolite classes. Mean fold change values of at least three independent experiments are shown for infection and control experiments with 95% confidence intervals (*t* test). The dashed lines correspond to |log_2_ FC| = log_2_ 1.5) (data from three biological replicates and two technical replicates). PC, phosphatidylcholine; PE, phosphatidylethanolamine; PS, phosphatidylserine.

THP-1 cells were infected at a multiplicity of infection (MOI) of 5, and immediate bacterial replication was comparable to replication in previous reports (39), based on CFUs that increased 3.6-fold between 4 and 48 h postinfection (h p.i.). To study metabolite dynamics during infection, we performed untargeted metabolomics analysis at 0, 4, 24, and 48 h p.i. (40). Based on the pilot experiment with bacterium–THP-1 cell mixtures, we expected measurements to reflect mainly host metabolic changes caused by either consumption of nutrients by the infecting bacteria or the response of host cells to phagocytosis. To control for the latter, we used gamma-irradiated tubercle bacilli, which still undergo phagocytosis and trigger a cellular response, but do not engage in active metabolism, i.e., consumption of nutrients. A total of 1,886 detected ions were annotated using the combined metabolite list described above, including 108 unique to mycobacteria and 173 unique to the lipids list (Fig. 2A and Table S1).

To identify metabolites associated with dynamic changes during infection, we applied a three-step procedure. First, we reduced measurement noise applying the permutation filtering method for dynamic data recently reported (44). This method is based on the assumption that “real” metabolic changes are a continuous process, so that data of neighboring time points show smaller variation than randomly picked data (permutation of time points). Second, for each time point, we performed comparative analyses of the metabolomes of infected and uninfected cells and defined the threshold for significant changes (|log_2_ fold change| > log_2_ 1.5; FDR < 0.05). Third, we applied a significance threshold for the comparison between the changes in the infection experiment versus control experiment (FDR < 0.05). These three subsequent steps led to the identification of consistently and significantly changing (132 increasing, 394 decreasing) metabolites during the 48 h of infection (Fig. 2B and Table S1). To discover general metabolic trends in the host upon *M. tuberculosis* infection, we performed a metabolite set enrichment analysis for both significantly increased and decreased metabolites using the functional set definitions of the Human Metabolome Database (HMDB) (45, 46).

Accumulating metabolites were significantly enriched for various subclasses of lipids, such as monoacyl glycerols (FDR = 0.05) and phosphatidylinositol phosphates (FDR = 5 × 10^−4^) (Fig. 2C and Table S1). This is in agreement with previous observation of increased lipid levels in macrophages upon ingestion of *M. tuberculosis* (47, 48). This lipid accumulation can be caused by shedding of bacterial lipids (49, 50), through the pathogen’s interference with the host’s lipid turnover (51), and by the activity of bacterial lipolytic enzymes digging carbons from host membranes (52). The latter mechanism may explain the observed accumulation of monoacyl glycerols and fatty acids at the expense of phosphatidylethanolamines, phosphatidylcholines, and phosphatidylserine that decreased during infection (Fig. 2D).

The major functional classes enriched among decreasing metabolites were amino acids (FDR = 0.3 × 10^−6^), nucleotides (FDR = 2 × 10^−3^), and sugar phosphates (FDR = 4 × 10^−4^) (Fig. 2C and Table S1). These compounds are potential mycobacterial substrates, some of which have already been reported, such as aspartate and asparagine (18, 19) (Fig. 2D). Moreover, a recent study based on ^13^C-tracing experiments proposed the uptake of various amino acids by intracellular *M. tuberculosis* (7). The availability of another potential carbon source is indicated by the enrichment for the chemical classes of monosaccharide and sugar phosphates (FDR = 4 × 10^−4^) (Fig. 2D). The uptake of phosphorylated carbohydrate(s) by intracellular mycobacteria is intriguing, because it provides not only carbon but also phosphorus, which appears to be limiting during infection (53). Overall, the metabolic analyses suggest that mycobacteria have access to a variety of nutrients inside the macrophage, such as amino acids, nucleotides, sugar phosphates, lipids, and fatty acids.

### Dynamic transcriptional changes in the host and pathogen during infection.

**FIG 3:**
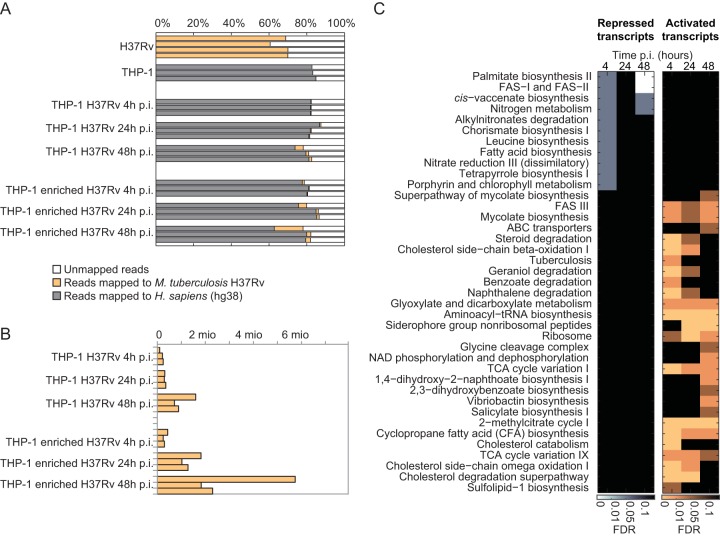
Analysis of changes in the mycobacterial transcriptome during infection. (A) RNA-seq read mapping statistics for all samples. (B) Total amount of reads (in millions) mapped to the *M*. *tuberculosis* H37Rv genome. (C) TBDB and KEGG metabolic pathway enrichment (displayed only if enrichment FDR was <0.1) of changing transcripts at each time point.

To obtain further insights into host-pathogen interactions, we investigated whether transcriptional adaptations of both host and pathogen during infection reflect multinutrient acquisition of the bacteria. Analogous to metabolomics, the transcriptome was analyzed at 0, 4, 24, and 48 h p.i. Complete transcriptome analysis was performed on rRNA-depleted total RNA by microbe-enriched dual RNA sequencing (dual RNA-seq) (54). The physical mycobacterial RNA enrichment yielded an increased number of reads for mycobacterial genes (up to 5.7 million) and hence, improved the statistical power of mycobacterial transcript measurements (Fig. 3A and B). Analyses of host transcripts were performed on samples without physical enrichment of mycobacterial RNA. RNA reads could be mapped to 4,008 mycobacterial annotated genes and 17,198 human annotated genes (Table S2).

10.1128/mSystems.00057-17.8 TABLE S2  Dual RNA-seq data of the THP-1 infection time course experiment. Pathway enrichment analysis results of both altered *M. tuberculosis* (Mtb) and human gene expression upon infection. Analysis of mycobacterial transporter expression during THP-1 infection. Download TABLE S2, XLSX file, 3.2 MB. Copyright © 2017 Zimmermann et al. 2017 Zimmermann et al. This content is distributed under the terms of the Creative Commons Attribution 4.0 International license.

Similar to the analysis of metabolite data, we aimed to identify general trends in the transcript changes. We first defined differentially expressed transcripts during infection (|log_2_ fold change| > log_2_ 1.5; FDR < 0.05 compared to uninfected samples) and performed pathway enrichment analysis using KEGG and TBDB pathway definitions (55). Upregulated host transcripts were mainly enriched for infection-associated pathways, such as antigen processing and presentation, for steroid and primary bile acid biosynthesis, and for xenobiotic degradation pathway. In contrast, aminoacyl-tRNA biosynthesis and several signaling pathways were among the significantly enriched functional groups of downregulated host transcripts (Table S2). In mycobacteria, several amino acid biosynthetic pathways were enriched among the downregulated transcripts, whereas glycine cleavage pathways and the previously reported methylcitrate cycle and steroid degradation were enriched in the group of upregulated transcripts (Fig. 3C and Table S2). Overall, transcriptional changes in the host revealed a general stress response, whereas numerous pathway activation and deactivation events in the pathogen indicated major mycobacterial adaptations during infection. Although pathway enrichment analysis provides a general picture of the changes occurring, it strongly depends on arbitrary pathway definitions and is performed separately for metabolites and genes. To combine these data to characterize host-pathogen interactions better, we next integrated metabolite and RNA-seq data into an unbiased genome-scale reaction pair network.

### Unbiased integration of metabolomics and RNA-seq data using a genome-wide reaction pair network.

**FIG 4:**
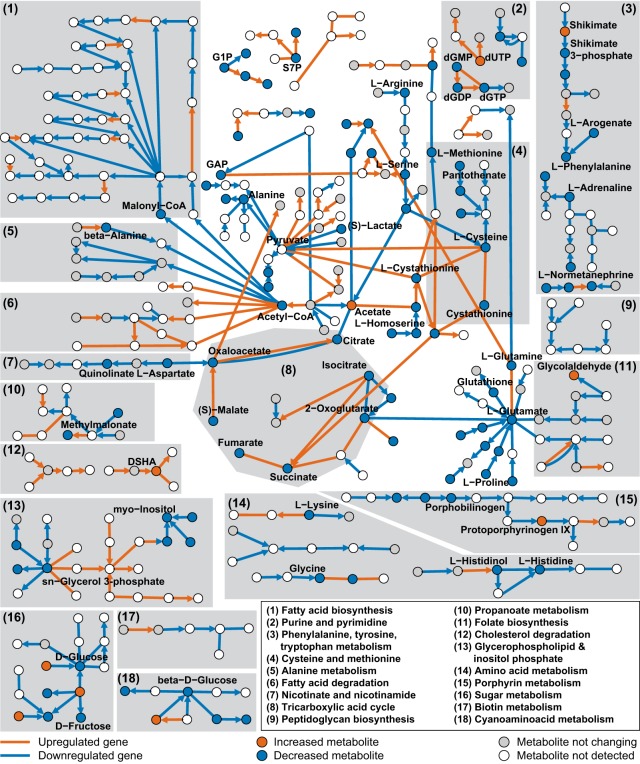
Systematic analysis of host-pathogen interactions. Results of genome-wide KEGG reaction pair network analysis summarized as a graph of metabolic reactions for which the corresponding gene activity is changing during infection (|log_2_ fold change| > log_2_ 1.5 and FDR < 0.05 for at least one time point p.i. [data from three biological replicates per time point]). Nodes represent metabolites, and edges represent reactions according to the KEGG main reaction pairs. Reaction directions are indicated according to the KEGG kgml pathway maps (the arrows do not necessarily correspond to reaction reversibility). Only metabolic paths connecting changing reactions with length of ≥3 are shown. G1P, glucose-1-phosphate; GAP, d-glyceraldehyde 3-phosphate; S7P, sedoheptulose 7-phosphate; DSHA, 4,5-9,10-diseco-3-hydroxy-5,9,17-tri-oxoandrosta-1(10),2-diene-4-oic acid. Upregulated genes had a log_2_ fold change > log_2_ 1.5 and FDR < 0.05 for at least one time point, and downregulated genes had a log_2_ fold change <−log_2_ 1.5 and FDR < 0.05 for at least one time point. Increased metabolites had a log_2_ fold change > log_2_ 1.5 and FDR < 0.05 for at least one time point, and decreased metabolites had a log_2_ fold change <−log_2_ 1.5 and FDR < 0.05 for at least one time point. Subgraphs were drawn with Cytoscape and manually classified into KEGG pathways (see Table S4 in the supplemental material).

To overlay metabolite and transcript data independent from *a priori* pathway definitions, we mapped the observed changes onto a genome-scale reaction pair network to identify changing metabolic subnetworks. We built the network based on KEGG reaction pairs and searched for subnetworks containing a minimum of three metabolically connected enzymes with significantly changing transcription (|log_2_ fold change| > log_2_ 1.5 and FDR < 0.05 for at least one time point), as recently applied to proteomics data (56). We identified 24 subnetworks of changing enzymes and metabolites with a length of 3 or more that represent metabolic changes upon infection (Fig. 4). Several subnetworks had an increasing (cholesterol degradation, nucleotide salvage pathway, and pentose phosphate pathway) or decreasing (aromatic and branched-chain aliphatic amino acids and porphyrin metabolism) transcriptional pattern, while some showed a mixed response (glycerophospholipids, fatty acids, and central carbon metabolism). In the following sections, we focus on the key host-pathogen interaction subnetworks identified.

### Cholesterol degradation fuels *M. tuberculosis* metabolism.

**FIG 5:**
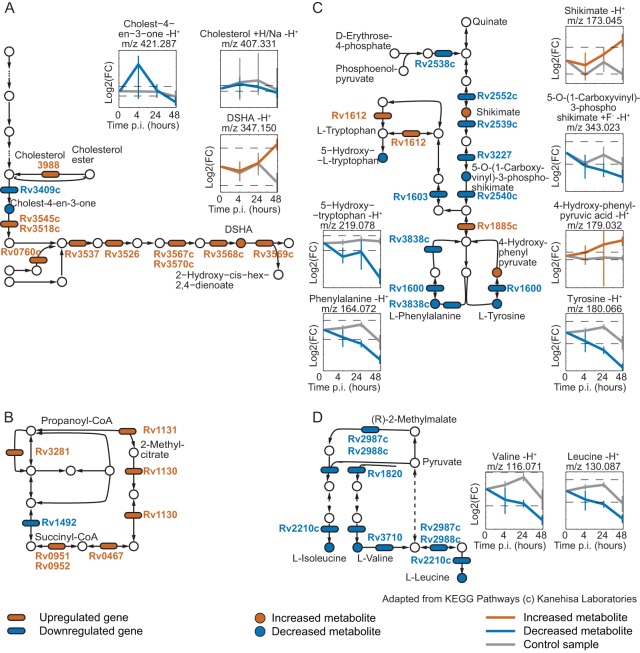
Metabolic pathway changes of intracellular *M*. *tuberculosis*. Detailed representation of the changes in selected subnetworks identified with genome-wide reaction pair analysis. Metabolites are indicated by circles, and genes are indicated by ovals. Red color indicates increase or upregulation (log_2_ FC ≥ log_2_ 1.5; *P* value < 0.05), and blue color indicates decrease or downregulation (log_2_ FC ≤−log_2_ 1.5; *P* value of <0.05); all data come from at least three biological replicates. Metabolite profiles show the mean fold change values from at least three independent experiments shown for infection (blue and red) and control (gray) experiments with 95% confidence intervals (*t* test). The dashed lines correspond to |log_2_ FC| = log_2_ 1.5. (A) Human cholesterol biosynthesis pathway and cholesterol catabolism in *M. tuberculosis*. (B) Decreasing levels of phenylalanine, tyrosine, and 5-hydroxytryptophan indicate potential uptake of these aromatic amino acids by intracellular *M. tuberculosis*, whereas their biosynthesis is downregulated. (C) The methylcitrate cycle feeding propanonyl-CoA into central carbon metabolism is transcriptionally activated during infection. (D) Decreasing levels of leucine/isoleucine and valine indicate potential uptake of these branched-chain amino acids by intracellular *M. tuberculosis*, whereas their biosynthesis is downregulated. The pathway maps are adapted from pathway maps in the KEGG Pathway database (42), with permission from the publisher.

Bacterial consumption and degradation of a host metabolite should lead to its decrease and to activation of the pathogen’s catabolic genes (activated metabolic subnetworks in Fig. 4). Cholesterol utilization by intracellular tubercle bacilli has been discovered through different experimental approaches (23, 34, 57) and hence served as an illustrative consumption example. Most bacterial genes of the catabolic pathway were expressed at higher levels during infection. Consistent with the hypothesis of induced catabolism, we observed accumulation of the intermediate 4,5-9,10-diseco-3-hydroxy-5,9,17-tri-oxoandrosta-1(10),2-diene-4-oic acid (DSHA), which is not produced by humans and hence directly reflects mycobacterial cholesterol degradation (Fig. 5A). Host cholesterol levels decreased by 80% (*P* = 0.09), and other cholesterol derivatives were also depleted during infection (Fig. 5A). As a consequence of such cholesterol depletion, host cells may need to replenish their cholesterol pools. In fact, we found induced expression of methylsterol monooxygenase 1 (EC 1.14.13.72) (MSMO1; Entrez gene identifier 6307) and the lipase A/cholesterol esterase (EC 3.1.1.13) (LIPA; Entrez gene identifier 3988) plus general enrichment of the steroid biosynthesis pathway among the host genes with increased expression during infection, which is consistent with increased cholesterol synthesis (Fig. 5A and Table S2).

Cholesterol degradation results in profound abundance of propanoyl-coenzyme A (CoA), which becomes toxic unless further metabolized (58, 59). Expectedly, we found strong transcriptional induction of the mycobacterial methylcitrate cycle, which converts propanoyl-CoA and oxaloacetate to succinate and pyruvate (Fig. 5B). Increased expression of Rv3280 and Rv3281 involved in converting propanoyl-CoA to methylmalonyl-CoA (Fig. 5B) indicated increased synthesis of methylmalonyl-CoA. This is likely linked to its role as a building block for branched-chain fatty acids, which have been found to be more abundant under axenic growth conditions on cholesterol (59). This is further supported by the fact that the expression of *mutA* (methylmalonyl-CoA mutase, Rv1492), converting methylmalonyl-CoA to succinyl-CoA in the tricarboxylic acid (TCA) cycle, was downregulated (Fig. 5B). These data underscore prior evidence of *in vivo* cholesterol consumption and indicate that the propanoyl-CoA produced by cholesterol degradation is converted to methylcitrate to fuel central carbon metabolism or to methylmalonyl-CoA to enter lipid biosynthesis (34).

Cholesterol degradation illustrates the pathogen’s activation of catabolic pathways to utilize host metabolites, which are consequently depleted in host cells. On the basis of a comparable activation pattern, we provide evidence that tubercle bacilli utilize glycine and sugar phosphates through the glycine cleavage and rhamnose biosynthesis pathways, respectively (Fig. S2A and B).

10.1128/mSystems.00057-17.2 FIG S2  Metabolic pathway changes of intracellular *M. tuberculosis*. (A) Glycine cleavage pathway in *M. tuberculosis*. Metabolites are indicated by circles, and genes are indicated by ovals. Red color indicates increase or upregulation (log_2_ FC ≥ log_2_ 1.5; *P* value < 0.05), and blue color indicates decrease or downregulation (log_2_ FC ≤−log_2_ 1.5; *P* value < 0.05); all data come from at least three biological replicates. For the metabolite traces, mean fold change values from at least three independent experiments are shown for the infection experiments (blue and red) and the control experiments (gray) with 95% confidence intervals (*t* test). The dashed lines correspond to |log_2_ FC| = log_2_ 1.5. Decreasing levels of glycine indicate that it is a potential carbon source of bacteria, whereas genes in the glycine cleavage pathway are upregulated. (B) Decreasing levels of sugar phosphates indicate potential uptake of these compounds by intracellular *M. tuberculosis*. Genes in the polyketide sugar biosynthesis pathway, which is fueled by sugar phosphates, are upregulated. Pathway maps are adapted from pathway maps in the TBDB database with permission from the publisher. (C) Decreasing levels of pyrimidine biosynthesis intermediates indicate potential uptake of these compounds by intracellular *M. tuberculosis*. Genes corresponding to the initial steps of *de novo* pyrimidine biosynthesis from the pentose phosphate pathway are downregulated, whereas genes coding several nucleotide salvage enzymes are upregulated. The pathway map was adapted from a pathway map in the KEGG database (pyrimidine biosynthesis) (42) with permission from the publisher. Download FIG S2, EPS file, 1.4 MB. Copyright © 2017 Zimmermann et al. 2017 Zimmermann et al. This content is distributed under the terms of the Creative Commons Attribution 4.0 International license.

### Consumption of hydrophobic amino acids deactivates anabolism by mycobacteria.

Among the deactivated mycobacterial subnetworks were nonpolar and aromatic amino acid biosynthesis pathways, i.e., expression of most genes involved in valine, leucine, and isoleucine biosynthesis as well as phenylalanine, tyrosine, and tryptophan biosynthesis was downregulated during infection (log_2_ fold change <– log_2_ 1.5; FDR < 0.05), whereas the levels of the corresponding end products decreased over time (log_2_ fold change <−log_2_ 1.5; FDR < 0.05) (Fig. 5C and D). This pattern of transcript and metabolite data suggests that these amino acids are directly utilized by *M. tuberculosis*, which is supported by a recent study demonstrating replication of a tryptophan auxotrophic *M. tuberculosis* strain in human monocyte-derived macrophages (20). As a consequence of supplemented biosynthesis with host-derived amino acids, corresponding anabolic pathways are downregulated. For other amino acids, expression patterns of biosynthetic and degradation pathways changed in different directions, suggesting that *M. tuberculosis* depends, at least in part, on their biosynthesis during infection. Nonpolar and aromatic amino acids may be particularly accessible nutrients for *M. tuberculosis* because their hydrophobic nature facilitates entry into the phagosome by passive membrane diffusion.

Another example of metabolite uptake that deactivates biosynthesis in intracellular mycobacteria is pyrimidine metabolism, since levels of pyrimidine intermediates decreased during infection, transcription of the enzymes required for *de novo* synthesis from the pentose phosphate pathway intermediates was also decreased, whereas enzymes of the nucleotide salvage pathway were increased (Fig. S2C). In contrast to catabolic cholesterol degradation, these examples illustrate the availability of biomass precursors at the site of infection, because bacterial consumption deactivates their biosynthetic pathways in *M. tuberculosis*.

### Integrating RNA-seq constraints into a genome-scale model of host-pathogen interactions.

Although data integration using a genome-wide reaction pair network identified subnetworks of particular interest during infection, this approach focused solely on the bacterial response without considering metabolic host-pathogen interactions in their entirety. To obtain a more integrated picture, we modeled metabolic fluxes between host and pathogen using a comprehensive combined genome-scale metabolic model of macrophages and intracellular tubercle bacilli (38). This model contains both human and mycobacterial metabolic networks with gene-protein-reaction annotations connected through the phagosomal compartment. We integrated the dual RNA-seq data of both host and bacterial gene expression into the model using the gene-protein-reaction annotations and estimated interspecies metabolic fluxes between the host and pathogen to gain a complete view of the infection system behavior.

Modeling metabolic host-pathogen interactions was performed in five steps. First, we expanded the existing model by including the reactions that we identified to be transcriptionally changed in the pathogen during infection and that were missing; i.e., adding cholesterol degradation, methylcitrate cycle and the capacity to synthesize glycerol for the production of glycerolipids. Second, we updated the gene-protein-reaction rules in the original model using the latest version of human (Recon 2) and mycobacterial models (60). Third, we relaxed the phagosomal uptake constraints in the existing model, which forced the bacteria to utilize glycerol and nitrogen monoxide as the major carbon source and sole nitrogen source, respectively. This step is justified by previous work showing that THP-1 cells do not produce prominent amounts of nitrogen monoxide (61) and that glycerol consumption plays a negligible role during infection (62). Fourth, we used an optimization procedure to find the flux solution in the modified model that maximizes either macrophage or mycobacterial biomass flux. The macrophage maximal biomass flux was 0.027 h^−1^ as in the original model (38), whereas the mycobacterial maximal biomass flux was 0.029 h^−1^ (compared to 0.0021 h^−1^ with the original constraints) given the possibility to take up different nutrients from the phagosome. This corresponds to an estimated mycobacterial doubling time of 24 h, which matches our experimental data based on CFUs indicating roughly two replications during the 48-h infection experiment. Fifth, following the rationale that gene expression and fluxes through an enzyme are loosely related, we used transcript data to formulate an optimization function based on activation and repression of genes, as previously described (63). Briefly, we searched for a flux solution through the network that maximizes the number of active and inactive reactions corresponding to increased and decreased gene expression levels, respectively, while satisfying stoichiometric constraints. This procedure results in condition-dependent flux solutions that mimic the metabolic behavior of the cells provided RNA-seq fold change data and thresholds as model inputs.

**FIG 6:**
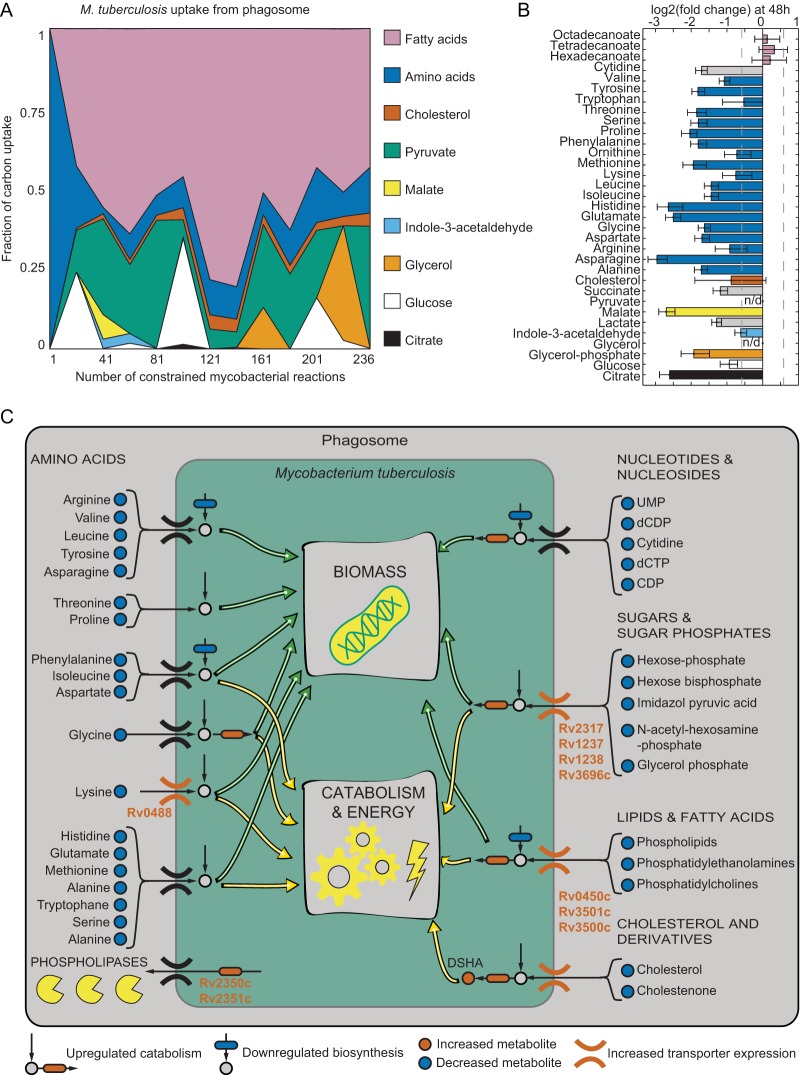
Genome-scale host-pathogen metabolic model analysis. (A) Sensitivity analysis of predicted nutrient fluxes between host and bacteria to the number of significantly changing transcripts used to constrain the genome-scale metabolic model. Flux values are normalized to the sum of bacterial uptake fluxes. (B) Fold changes of metabolites predicted to be bacterial substrates by constraint-based modeling. Values are given in log_2_ FC of 48 h p.i. to 0 h (uninfected THP-1 cells) (data for at least three biological replicates). Significantly decreasing metabolites (FDR < 0.05) are depicted in blue. (C) Graphical summary of the *in silico* simulations of the metabolic host-pathogen interactions and the transcriptional analysis of bacterial transporters illustrating bacterial use of multiple energy and carbon sources during infection. The underlying experimental evidence is demonstrated by the colored symbols depicted.

In the host-pathogen model, bacteria are enabled to consume 68 different substrates, based on reported substrates under *in vitro* culture conditions (38). In order to investigate whether the RNA-seq data can provide insights into early host-pathogen interactions, we performed sensitivity analysis of the predicted phagosomal uptake to the mycobacterial genes used in the optimization function. We varied the number of differentially expressed genes used to formulate the flux optimization function by changing the fold change threshold and obtained the optimal flux solution for each set of the changing genes. Constrained by the RNA-seq data, the identified flux solution indicated consumption of 33 of the 68 possible *M. tuberculosis* nutrients for at least one of the tested sets of changing genes. Whereas cholesterol and aspartate were predicted to be taken up from the phagosome for >85% of the tested gene sets in the sensitivity analysis, glycerol was utilized rarely (in solutions for <30% tested gene sets), which is in agreement with previous experimental findings (62) (Fig. 6A). To assess the temporal development of the host-pathogen interactions, we also compared the solutions resolved for the constraints of each time point separately. As the infection progresses, fatty acids seem to become a less prominent carbon source, whereas amino acids gain importance as a carbon source (Fig. S3).

10.1128/mSystems.00057-17.3 FIG S3  Temporal analysis of predicted nutrient fluxes between host and bacteria in the genome-scale metabolic model. To obtain the flux distribution, up- and downregulated transcripts were used to constrain the optimization function in the iMAT procedure (|log_2_ FC| > log_2_ 1.5 and FDR < 0.05 at the corresponding time point). No constraints were used for the biomass fluxes. Flux values are normalized by the sum of bacterial uptake fluxes. Download FIG S3, EPS file, 0.7 MB. Copyright © 2017 Zimmermann et al. 2017 Zimmermann et al. This content is distributed under the terms of the Creative Commons Attribution 4.0 International license.

In order to investigate the contribution of the predicted substrate consumption on mycobacterial growth, we repeated the flux optimization procedure using the full list of RNA-seq constraints (all genes that pass the threshold of |log_2_ fold change| > log_2_ 1.5 and FDR < 0.05) for each set of constraints on the lower bound of mycobacterial and host cell biomass flux, ranging from 0 to 50% of either maximal value (36 biomass constraint sets in total). For all parameter sets, fatty acids, amino acids, and cholesterol were predicted to be consumed (Fig. S4), indicating the robustness of the predictions against the set biomass parameter in the model. The consumption profiles, however, varied quantitatively, depending on the set constraints (Fig. S5). For each of the 36 constraint sets, we calculated the fraction of consumed metabolite used for biomass formation through comparison of uptake and biomass flux multiplied by the corresponding coefficient (Fig. S6). Among 33 identified carbon sources, 9 were used mostly for biomass (fraction > 0.50), whereas the remaining ones are mostly catabolized to produce other metabolites or energy (fraction < 0.5) (Table 1 and Table S3). The main nitrogen sources were amino acids rather than nitrogen monoxide, consistent with increased gene expression of Rv2220 catalyzing the central nitrogen metabolism reaction from ammonia and glutamate to glutamine (Table S2). Of the 33 predicted carbon sources, 31 could be detected by our metabolomics measurements, of which 26 were significantly decreased during infection (log_2_ fold change < −log_2_ 1.5; FDR < 0.05; Fig. 6B and Table S1), supporting our hypothesis of their bacterial consumption.

10.1128/mSystems.00057-17.4 FIG S4  Activity of predicted nutrient fluxes between host and bacteria in the genome-scale metabolic model to the constraints on biomass fluxes. Heatmaps represent the fraction of each group of nutrients among the predicted mycobacterial carbon uptake fluxes. Each solution was obtained by the iMAT procedure including up- and downregulated transcripts (|log_2_ FC| > log_2_ 1.5 and FDR < 0.05 for at least one time point). The 36 sets of constraints on the mycobacterial biomass function (*x* axes) and host biomass function (*y* axes) were tested. Flux values are normalized by the sum of bacterial uptake fluxes. Download FIG S4, EPS file, 0.9 MB. Copyright © 2017 Zimmermann et al. 2017 Zimmermann et al. This content is distributed under the terms of the Creative Commons Attribution 4.0 International license.

10.1128/mSystems.00057-17.5 FIG S5  Sensitivity of predicted nutrient fluxes between host and bacteria in the genome-scale metabolic model to the constraints on biomass fluxes. To obtain the flux distribution, up- and downregulated transcripts were used to constrain the optimization function in the iMAT procedure (|log_2_ FC| > log_2_ 1.5 and FDR < 0.05 for at least one time point). (A) Two constraint values on THP-1 biomass flux with a flexible constraint on the mycobacterial biomass flux. (B) Two constraint values on mycobacterial biomass flux with a flexible constraint on the THP-1 biomass flux. Flux values are normalized by the sum of bacterial uptake fluxes. Download FIG S5, EPS file, 1.3 MB. Copyright © 2017 Zimmermann et al. 2017 Zimmermann et al. This content is distributed under the terms of the Creative Commons Attribution 4.0 International license.

10.1128/mSystems.00057-17.6 FIG S6  Analysis of nutrient fractions used for biomass formation depending on the constraints on the genome-scale host-pathogen metabolic model. To obtain the flux distribution, up- and downregulated transcripts were used to constrain the optimization function in the iMAT procedure (|log_2_ FC| > log_2_ 1.5 and FDR < 0.05 for at least one time point). Each FBA (flux balance analysis) parameter set is represented with a pair of constraints on the THP-1 and mycobacterial biomass fluxes. The biomass fraction is calculated by dividing the nutrient uptake flux by the nutrient contribution to the corresponding biomass flux in the bacterium. Download FIG S6, EPS file, 1.2 MB. Copyright © 2017 Zimmermann et al. 2017 Zimmermann et al. This content is distributed under the terms of the Creative Commons Attribution 4.0 International license.

10.1128/mSystems.00057-17.9 TABLE S3  Modified host-pathogen genome-scale model. *M. tuberculosis* substrate predictions based on mRNA-constrained flux balance analysis and gene essentiality analysis. Download TABLE S3, XLSX file, 1 MB. Copyright © 2017 Zimmermann et al. 2017 Zimmermann et al. This content is distributed under the terms of the Creative Commons Attribution 4.0 International license.

10.1128/mSystems.00057-17.10 TABLE S4  Genome-wide KEGG reaction pair network analysis results represented as a list of graph edges (metabolic reactions) corresponding to Fig. 4. Download TABLE S4, XLSX file, 3.9 MB. Copyright © 2017 Zimmermann et al. 2017 Zimmermann et al. This content is distributed under the terms of the Creative Commons Attribution 4.0 International license.

**TABLE 1:** Average fractions of the metabolites taken up from the phagosome used for biomass synthesis^a^

Metabolite	Uptake frequency in solutions	Mean biomass fraction
Cholesterol	1.00	0.00
Octadecanoate	1.00	0.00
Tetradecanoate	1.00	0.07
Hexadecanoate	1.00	0.27
Cytidine	0.97	0.00
Glucose	0.97	0.11
Leucine	0.87	0.82
Arginine	0.87	0.89
Glutamate	0.80	0.62
Pyruvate	0.77	0.00
Isoleucine	0.77	0.37
Aspartate	0.73	0.03
Ornithine	0.70	0.00
Serine	0.63	0.08
Methionine	0.53	0.19
Glycerol	0.50	0.00
Indole-3-acetaldehyde	0.40	0.00
Threonine	0.40	1.00
Citrate	0.37	0.00
Lysine	0.37	0.06
Phenylalanine	0.33	0.54
Valine	0.30	0.73
Histidine	0.20	0.04
Malate	0.17	0.00
Asparagine	0.17	1.00
Proline	0.13	1.00
Glycerol-3-phosphate	0.10	0.00
Succinate	0.10	0.00
Tryptophan	0.10	0.02
Glycine	0.10	0.69
Lactate	0.07	0.00
Alanine	0.07	0.15
Tyrosine	0.03	0.00

a Average fractions of the metabolites taken up from the phagosome used for biomass synthesis predicted by flux balance analysis with RNA-seq constraints. The fraction is an average of 36 model runs with different parameter sets constraining the lower bounds of mycobacterial and THP-1 biomass fluxes.

Overall, *M. tuberculosis* appears to follow a multiple-substrate fueling strategy during the infection of macrophages, utilizing a large variety of different nutrients ranging from amino acids to carbohydrates and lipids (Fig. 6). This conclusion is further supported by the activation of several mycobacterial transporters of sugars and amino acids (Fig. 6C and Table S2). Compared to a single carbon source metabolism, this metabolic adaptation suggests increased flexibility and hence robustness of *M. tuberculosis* toward interventions, either through host responses or chemotherapeutic treatments.

## DISCUSSION

We characterized metabolic interactions between human macrophage-like cells and *M. tuberculosis* during early infection by integrating dynamic metabolomics and dual RNA-seq data with two genome-wide network analysis approaches. In the first approach, we identified metabolic subnetworks that were differentially active during infection of human macrophage-like cells with *M. tuberculosis*. This genome-wide reaction pair analysis revealed two distinct patterns of activity employed by intracellular tubercle bacilli to utilize available host metabolites. For certain consumed host metabolites, the pathogen activates specific metabolic pathways to make use of newly acquired substrates, such as the cholesterol degradation, methylcitrate cycle, and glycine cleavage pathways. Other metabolites, such as some amino acids and nucleotides, are directly used for anabolism, allowing *M. tuberculosis* to downregulate respective endogenous biosynthesis pathways. In addition to changes in mycobacterial metabolism, we identified adaptations in host metabolism and found indications for mycobacterial manipulation of host metabolism, for example an altered lipid composition that was likely caused by upregulated mycobacterial lipases during infection. In the second approach, the key findings of the first network analysis built the basis for refinements of a metabolic host-pathogen model. Subsequent genome-scale modeling with RNA-seq-derived constraints of the metabolic interactions between macrophages and intracellular tubercle bacilli revealed a multiple-nutrient strategy during early infection, utilizing more than 30 different carbon and nitrogen sources. Sensitivity analysis of the set constraints further weighted the robustness of each predicted substrate. Metabolic flux analysis further enabled us to estimate the relative contribution of each nutrient to the pathogen’s intracellular growth and survival at different time points of the infection through determination of the fates of these nutrients in either anabolism or catabolism to serve as biomass precursors or energy sources, respectively.

The demonstrated data-driven modeling of the host-pathogen interactions serves as a platform for future experiments aiming at integrating multi-omics data during bacterial infections. *M. tuberculosis* infection cycles can span decades during which pathogen and host undergo different stages as a result of persistent interactions (64). Generally, *in vitro* infection models such as the early infection model used here cannot recapitulate this complexity and are subject to inherent heterogeneity with respect to infection levels and intracellular growth. The results thus have to be interpreted with appropriate caution. Nevertheless, the results may give insights into some general principles of mycobacterial infection strategies. The multiple-substrate fueling of intracellular *M. tuberculosis* demonstrated here underlines the organism’s ability to utilize even sparsely available nutrients. This ability renders *M. tuberculosis* robust against pharmacological or host interventions that aim at blocking the food supply, but our findings could potentially help to identify the pathogen’s “metabolic Achilles heel” for future therapeutic intervention measures.

To illustrate how our and future data may be used for guidance of therapeutic interventions, we performed a gene essentiality analysis with the combined host-pathogen genome-wide metabolic model before and after integration of our omics data by setting the flux value for each mycobacterial reaction to zero and estimating bacterial *in vivo* growth by solving the mycobacterial biomass optimization problem. The suggested multisubstrate strategy reduced the predicted essential metabolic genes from 162 to 103 (growth rate reduction > 90% compared to the wild type). The 59 genes that became “nonessential” under conditions of multisubstrate availability belonged mostly to amino acid metabolism (44 genes) (Table S3). Of these 59 genes, 18 were genes that were also found to be nonessential under axenic culture conditions (65). The 103 genes that were predicted to impact mycobacterial *in vivo* replication under multisubstrate utilization conditions were mainly found in membrane (27 genes), amino acid (17 genes), nucleic acid (29 genes), and lipid metabolism (18 genes). Our results suggest that there are fewer genes truly essential for mycobacteria during infection, thus narrowing down options of potential drug targets in mycobacterial metabolism. Supported by the recently discovered growth inhibitors of intracellular *M. tuberculosis* targeting cholesterol metabolism (57), we believe that our identified active metabolic subnetworks should be focused upon in future investigations aiming at identifying suitable metabolic perturbation targets in *M. tuberculosis* infection.

## MATERIALS AND METHODS

### Infection of the human macrophage-like cell line THP-1.

The THP-1 cell line (ATCC TIB-202; American Type Culture Collection) was maintained in Roswell Park Memorial Institute medium 1640 supplemented with 10% (vol/vol) fetal calf serum, 2 mM glutamine, 1 mM sodium pyruvate, and 0.05 mM 2-mercaptoethanol in a humidified 5% carbon dioxide atmosphere at 37°C. An estimated 5 × 10^6^ cells/well in a six-well plate were differentiated for 24 h using culture medium containing 40 ng/ml phorbol 12-myristate 13-acetate. The cells were then washed with fresh culture medium and incubated for 48 h. Exponentially grown *M. tuberculosis* H37Rv (ATCC 27294; American Type Culture Collection) was pelleted (3,200 rpm, room temperature [RT], 10 min), washed twice with phosphate-buffered saline (PBS), and resuspended in THP-1 culture medium. THP-1 cells were infected at a multiplicity of infection (MOI) of 5. For mock infections, gamma-irradiated *M. tuberculosis* H37Rv was used (catalog no. NR-49098; BEI Resources). The *M. tuberculosis* culture medium suspension was added to the differentiated cells and subsequently incubated for 4 h. The infection mix was removed, and the cells were washed twice with prewarmed PBS and incubated further using fresh medium. RNA isolation and metabolite extraction were performed at designated time points postinfection (p.i.).

### Metabolic sample preparation, measurements, and data analysis.

Cells were washed twice with prewarmed ammonium acetate solution (75 mM, pH 7.5), frozen on dry ice, and stored at −80°C until further processing. Metabolites were extracted three times with 70% ethanol at 78°C for 2 min, and the three extracts were pooled, dried under vacuum at ambient temperature (22°C), and resuspended in 0.5-ml water for metabolic analysis. Untargeted measurements of metabolites was performed by direct injection mass spectrometry using a quadrupole time of flight instrument (Agilent 6550 Q-TOF) with the following settings: negative mode; 4-GHz high-resolution mode; scanning the *m/z* range of 50 to 1,000 following published protocol (40). Negative ionization mode was chosen for its ability to detect the organic acids and phosphorylated compounds central to carbon metabolism and hence essential for the employed metabolic models. Ions were annotated to metabolites based on exact mass considering [M-H+] and [M+F-] ions using the metabolite reference list compiled from the Kyoto Encyclopedia of Genes and Genomes (KEGG) metabolite repositories hsa (*Homo sapiens*) and mtu (*M. tuberculosis*), the Human Metabolome Database (HMDB) (46), and Mtb Lipid Database and MycoMass databases of mycobacterial lipids. Ions were assigned to metabolites using this list and allowing a mass tolerance of 1 mDa and an intensity cutoff of 1,500 counts as previously described (40). Raw intensity values were normalized by mean intensity value in each sample. The annotation was filtered in three steps. (i) For each ion, only metabolites with the top annotation score were retained. (ii) For each metabolite, only the annotation with the top score was retained. (iii) The annotations with adducts such as NaCl, H/Na, H/K were removed. Measurements were filtered to account for noise using the permutation filtering method recently reported (44). In brief, the variability of neighboring time point measurements was calculated and compared to the variability of the time point measurements arranged in a randomized order. The measurements with variability indifferent from the one of randomized data were removed from further analysis (quantile cutoff > 0.33).

### RNA isolation, mycobacterial RNA enrichment, and RNA sequencing.

Total RNA from mycobacterial cultures was prepared as previously described (66). Extraction of total RNA from THP-1 cells was prepared with TRIzol reagent using glycogen as a carrier according to the supplier’s recommendation (Life Technologies). Total RNA from *M. tuberculosis*-infected THP-1 cells without enrichment was isolated by abrasive particles in a reciprocal shaker with TRIzol (66). Enrichment of mycobacteria from infected THP-1 cells was carried out by differential lysis of host and mycobacterial cells using guanidine thiocyanate (GITC).

Infected cells were washed with PBS at room temperature (RT). Cold 4 M GITC was added to the monolayer, and the cells were transferred to a 1.5-ml screw-cap tube. After centrifugation, the pellet was resuspended in residual GITC and mixed with 1 ml TRIzol containing 20 µg/ml linear acrylamide, followed by incubation for 5 min at RT. Bacteria were disrupted by bead beating (FastPrep instrument; two cycles of 30 s at maximum speed with cooling on ice between cycles). The sample was centrifuged for 1 min at 4°C and 13,000 rpm, and the supernatant was transferred to a 2-ml screw-cap tube containing 200 µl chloroform, mixed, and incubated at RT for 5 min. After centrifugation at 4°C and 13,000 rpm for 10 min, RNA was extracted from the aqueous phase using the Qiagen RNeasy minikit including an on-column DNase digestion (Qiagen). The quality and quantity of total RNA were assessed using an Agilent 2100 bioanalyzer (Agilent Technologies) and a NanoDrop 1000 spectrophotometer.

Bar-coded RNA-seq libraries were prepared according to the *TruSeq RNA Sample Preparation v2 Guide* (67) without fragmentation, as no additional fragmentation step was required because the average final library size was approximately 350 bp, and without size selection. The Gram-positive bacteria Ribo-Zero (Epicentre) rRNA magnetic removal kit was used to remove bacterial rRNA from mycobacterial total RNA. For total RNA from uninfected THP-1 cells at time point 0 h, the Ribo-Zero magnetic kit human/mouse/rat was used, while depletion of rRNA from infections with and without enrichment were depleted with the Ribo-Zero magnetic gold kit (epidemiology). All cDNA libraries were verified for size distribution and quality using the highly sensitive DNA kit (Agilent) on a 2100 bioanalyzer and quantified with the Qubit 2.0 fluorometer (Life Technologies). Libraries from each consecutive experiment were pooled as 8-plex and on-board loaded with a Hi-Seq 1500 instrument. All sequencing reactions were carried out as rapid runs using TruSeq rapid PE (paired-end) cluster kits and a 200-cycle TruSeq rapid SBS (sequencing by synthesis) kit as 2 × 51 cycles including 7-cycle indexing in order to obtain 50-bp paired-end reads.

### Mapping of RNA-seq reads.

Raw reads were trimmed using Trimmomatic (68) to remove low-quality bases and adapters and quality checked using FastQC (http://www.bioinformatics.babraham.ac.uk/projects/fastqc/). Fastq files were imported in pairs into the CLC Genomics Workbench (CLC Bio, version 7.5.1) running on a CLC Genomics Server (CLC Bio, version 6.5.2). The imported reads were mapped to the mycobacterial genome H37Rv (NC_000962) and *Homo sapiens* genome hg38 (GRCh38) with the RNA-seq tool. For transporter analysis, RNA-seq data were mapped to a set of mycobacterial transporters compiled from the Transporter Classification Database (TCDB) [http://www.tcdb.org/download.php, section Retrieve protein FASTA sequence(s) with Organism Name, query transporters for mycobacterium]. The following parameters were used for mapping: mismatch cost of 2, insertion cost of 3, deletion cost of 3, length fraction of 0.8, similarity fraction of 0.8, maximum number of hits for a read of 10, and failed reads were removed. Mapping reports were generated for each sample and exported from the CLC Genomics Workbench in csv format for further analysis. Unique read counts were normalized by a size factor calculated for each sample as the median of the ratios of the detected gene counts to the counts of an averaged sample. The averaged sample was calculated as the geometric mean across all samples (69). Enriched samples were used for mycobacterial RNA analysis, and nonenriched samples were used for human RNA analysis.

### Statistical analysis.

Mass spectrometry and dual RNA-seq data were imported into Matlab (MathWorks) for quantitative and statistical analysis. For differential analysis, human samples with spiked bacteria at a ratio of 0, 5, 10, or 20 bacteria per human cell were compared to the human samples without bacteria using *t* test with unequal variances (four biological replicates and two technical replicates per condition). Metabolomics mixed samples from infection and control experiments at 4, 24, and 48 h p.i. were compared to uninfected human samples using a *t* test with unequal variances (three or four biological replicates and two technical replicates per time point). Fold changes of the infection versus control experiments at 4, 24, and 48 h p.i. were compared using a *t* test with unequal variances. RNA-seq nonenriched mixed samples at 4, 24, and 48 h p.i. were compared to the uninfected human samples; RNA-seq enriched mixed samples were compared to the mycobacterial samples using a negative binomial model (Matlab function nbintest with constant variance link and pooled/nonpooled variance for enriched/nonenriched samples) (three biological replicates per time point). *P* values were corrected for multiple hypotheses testing by calculating the false-discovery rate (FDR) by the Benjamini-Hochberg procedure. For functional group and pathway enrichment, metabolite classes were defined as in the HMDB database. Pathway descriptions were downloaded from KEGG and TBDB databases. Metabolites passing the threshold of |log_2_ fold change| > log_2_ 1.5, FDR < 0.05, and FDR(change to control) < 0.05 and transcripts passing the threshold of |log_2_ fold change| > log_2_ 1.5 and FDR < 0.05 were ranked according to *P* value or fold change at each time point. Enrichment analysis was performed with the Fisher exact test for each subset of size varying from 1 to the total changing set size, and the smallest *P* value was retained for each pathway, as proposed in the gene set enrichment analysis (GSEA) method (45). *P* values for the enrichment analysis were adjusted for multiple hypotheses testing by calculating FDR by the Benjamini-Hochberg procedure.

### Computational analysis.

All computational analyses were done in Matlab (MathWorks), if other tools are not mentioned explicitly. For KEGG reaction pair network analysis, the KEGG reaction pair list, KEGG reaction pair-mycobacterial gene association list, and KEGG compound list were downloaded from KEGG API (application programming interface) (http://rest.kegg.jp/). An in-house script in Python (Python 2.7) was used to retain only main reaction pairs. For each mycobacterial gene, a list of metabolites involved in reactions associated with this gene was created. A gene-gene connectivity matrix was built based on the metabolites associated with genes (if two genes are associated with reactions sharing a metabolite, they are connected through this metabolite). K-shortest path script based on Yen’s algorithm (http://www.mathworks.com/matlabcentral/fileexchange/32513-k-shortest-path-yen-s-algorithm) was used to calculate the shortest paths between two genes based on the connectivity matrix. All paths of length three and more were visualized in Cytoscape software (Cytoscape 2.8.3). Reaction directions were downloaded from KEGG (kgml pathway files) with an in-house Matlab script.

Flux balance analysis with RNA-seq constraints: the joint macrophage-mycobacteria model was downloaded from the repository (http://systemsbiology.ucsd.edu/InSilicoOrganisms/MacTB) and extended with metabolites and reactions listed in Table S3 in the supplemental material. The latest releases of human genome-scale model Recon 2.04 and iNJ661 (http://bigg.ucsd.edu/models/iNJ661) were used to update the gene-protein-reaction (GPR) information (Table S3). Flux balance analysis was performed optimizing for biomass production using the cplexlp tool from CPLEX Optimizers 12.5.1 (IBM). For RNA-seq data integration, reactions associated with human and mycobacterial transcripts passing the threshold of |log_2_ fold change| > log_2_ 1.5 and FDR < 0.05 for at least one time point were defined active or inactive. Mixed-integer linear programming (MILP) minimizing the flux through inactive reactions and maximizing the flux through active reactions was defined as described in reference 63 and solved using the cplexmilp tool. To perform sensitivity analysis with the RNA-seq constraints, mycobacterial genes were sorted by maximum absolute fold change, and all sets of parameters from 1 to the total number of changing model genes (236 genes) were used to constrain the model without additional biomass constraints. To perform sensitivity analysis with biomass constraints, a set of 36 constraints was used (all combinations of lower bounds for mycobacterial and TPH1 biomass fluxes from the set [0 10% 20% 30% 40% 50%] of maximum value). All changing genes were used to constrain the model. Based on the flux solution, catabolic flux of a metabolite was calculated as the difference between the phagosomal uptake flux and biomass requirements defined in the model for each set of biomass constraints, and the average fraction of metabolite taken up that was used for biomass formation was calculated. For the gene essentiality analysis, the original and modified models (with phagosomal uptake upper bounds for metabolites identified from sensitivity analysis to RNA-seq constraints set at 1,000) were used to solve the mycobacterial biomass optimization task with single gene knockout simulations. The genes were defined essential if the knockout resulted in more than 90% decreased growth rate compared to that of the wild-type strain.

### Accession number(s).

Raw sequence reads of the RNA-seq analysis can be downloaded from the EMBL-EBI European Nucleotide Archive under accession no. E-MTAB-5287. Metabolomics data are available in Table S1. The modified metabolic model and the browsable Cytoscape file of Fig. 4 can be downloaded from the laboratory’s webpage (http://www.imsb.ethz.ch/research/sauer.html). All numerical data used for all main and supplemental figures are provided in the supplemental tables.

## References

[1] Abu Kwaik Y, Bumann D 2013 Microbial quest for food in vivo: “nutritional virulence” as an emerging paradigm. Cell Microbiol 15:882–890. doi:10.1111/cmi.12138. 23490329

[2] Brown SA, Palmer KL, Whiteley M 2008 Revisiting the host as a growth medium. Nat Rev Microbiol 6:657–666. doi:10.1038/nrmicro1955. 18679171 PMC3115587

[3] Eisenreich W, Dandekar T, Heesemann J, Goebel W 2010 Carbon metabolism of intracellular bacterial pathogens and possible links to virulence. Nat Rev Microbiol 8:401–412. doi:10.1038/nrmicro2351. 20453875

[4] Fuchs TM, Eisenreich W, Heesemann J, Goebel W 2012 Metabolic adaptation of human pathogenic and related nonpathogenic bacteria to extra- and intracellular habitats. FEMS Microbiol Rev 36:435–462. doi:10.1111/j.1574-6976.2011.00301.x. 22092350

[5] Jamshidi N, Palsson BØ 2007 Investigating the metabolic capabilities of Mycobacterium tuberculosis H37Rv using the in silico strain iNJ661 and proposing alternative drug targets. BMC Syst Biol 1:26. doi:10.1186/1752-0509-1-26. 17555602 PMC1925256

[6] Ehrt S, Rhee K, Schnappinger D 2015 Mycobacterial genes essential for the pathogen’s survival in the host. Immunol Rev 264:319–326. doi:10.1111/imr.12256. 25703569 PMC4339221

[7] Beste DJV, Nöh K, Niedenführ S, Mendum TA, Hawkins ND, Ward JL, Beale MH, Wiechert W, McFadden J 2013 13.C-flux spectral analysis of host-pathogen metabolism reveals a mixed diet for intracellular Mycobacterium tuberculosis. Chem Biol 20:1012–1021. doi:10.1016/j.chembiol.2013.06.012. 23911587 PMC3752972

[8] Abu Kwaik Y, Bumann D 2015 Host delivery of favorite meals for intracellular pathogens. PLoS Pathog 11:e1004866. doi:10.1371/journal.ppat.1004866. 26110434 PMC4482385

[9] Schmidt F, Völker U 2011 Proteome analysis of host-pathogen interactions: investigation of pathogen responses to the host cell environment. Proteomics 11:3203–3211. doi:10.1002/pmic.201100158. 21710565

[10] Waddell SJ, Butcher PD 2007 Microarray analysis of whole genome expression of intracellular Mycobacterium tuberculosis. Curr Mol Med 7:287–296. doi:10.2174/156652407780598548. 17504113 PMC3123378

[11] Westermann AJ, Gorski SA, Vogel J 2012 Dual RNA-seq of pathogen and host. Nat Rev Microbiol 10:618–630. doi:10.1038/nrmicro2852. 22890146

[12] Kentner D, Martano G, Callon M, Chiquet P, Brodmann M, Burton O, Wahlander A, Nanni P, Delmotte N, Grossmann J, Limenitakis J, Schlapbach R, Kiefer P, Vorholt JA, Hiller S, Bumann D 2014 Shigella reroutes host cell central metabolism to obtain high-flux nutrient supply for vigorous intracellular growth. Proc Natl Acad Sci U S A 111:9929–9934. doi:10.1073/pnas.1406694111. 24958876 PMC4103312

[13] Kreibich S, Hardt W-D 2015 Experimental approaches to phenotypic diversity in infection. Curr Opin Microbiol 27:25–36. doi:10.1016/j.mib.2015.06.007. 26143306

[14] World Health Organization 2017 Tuberculosis fact sheet. World Health Organization, Geneva, Switzerland.

[15] Leemans JC, Juffermans NP, Florquin S, van Rooijen N, Vervoordeldonk MJ, Verbon A, van Deventer SJ, van der Poll T 2001 Depletion of alveolar macrophages exerts protective effects in pulmonary tuberculosis in mice. J Immunol 166:4604–4611. doi:10.4049/jimmunol.166.7.4604. 11254718

[16] O’Garra A, Redford PS, McNab FW, Bloom CI, Wilkinson RJ, Berry MPR 2013 The immune response in tuberculosis. Annu Rev Immunol 31:475–527. doi:10.1146/annurev-immunol-032712-095939. 23516984

[17] Vergne I, Chua J, Singh SB, Deretic V 2004 Cell biology of Mycobacterium tuberculosis phagosome. Annu Rev Cell Dev Biol 20:367–394. doi:10.1146/annurev.cellbio.20.010403.114015. 15473845

[18] Gouzy A, Larrouy-Maumus G, Bottai D, Levillain F, Dumas A, Wallach JB, Caire-Brandli I, de Chastellier C, Wu T-D, Poincloux R, Brosch R, Guerquin-Kern J-L, Schnappinger D, Sório de Carvalho LP, Poquet Y, Neyrolles O 2014 Mycobacterium tuberculosis exploits asparagine to assimilate nitrogen and resist acid stress during infection. PLoS Pathog 10:e1003928. doi:10.1371/journal.ppat.1003928. 24586151 PMC3930563

[19] Gouzy A, Larrouy-Maumus G, Wu TD, Peixoto A, Levillain F, Lugo-Villarino G, Guerquin-Kern JL, Gerquin-Kern JL, de Carvalho LP, Poquet Y, Neyrolles O 2013 Mycobacterium tuberculosis nitrogen assimilation and host colonization require aspartate. Nat Chem Biol 9:674–676. doi:10.1038/nchembio.1355. 24077180 PMC3856356

[20] Zhang YJ, Reddy MC, Ioerger TR, Rothchild AC, Dartois V, Schuster BM, Trauner A, Wallis D, Galaviz S, Huttenhower C, Sacchettini JC, Behar SM, Rubin EJ 2013 Tryptophan biosynthesis protects mycobacteria from CD4 T-cell-mediated killing. Cell 155:1296–1308. doi:10.1016/j.cell.2013.10.045. 24315099 PMC3902092

[21] Chang JC, Miner MD, Pandey AK, Gill WP, Harik NS, Sassetti CM, Sherman DR 2009 igr genes and Mycobacterium tuberculosis cholesterol metabolism. J Bacteriol 191:5232–5239. doi:10.1128/JB.00452-09. 19542286 PMC2725594

[22] Johansen KA, Gill RE, Vasil ML 1996 Biochemical and molecular analysis of phospholipase C and phospholipase D activity in mycobacteria. Infect Immun 64:3259–3266. 8757862 10.1128/iai.64.8.3259-3266.1996 PMC174216

[23] Pandey AK, Sassetti CM 2008 Mycobacterial persistence requires the utilization of host cholesterol. Proc Natl Acad Sci U S A 105:4376–4380. doi:10.1073/pnas.0711159105. 18334639 PMC2393810

[24] Lee W, VanderVen BC, Fahey RJ, Russell DG 2013 Intracellular Mycobacterium tuberculosis exploits host-derived fatty acids to limit metabolic stress. J Biol Chem 288:6788–6800. doi:10.1074/jbc.M112.445056. 23306194 PMC3591590

[25] McKinney JD, Höner zu Bentrup K, Muñoz-Elías EJ, Miczak A, Chen B, Chan WT, Swenson D, Sacchettini JC, Jacobs WR, Russell DG 2000 Persistence of Mycobacterium tuberculosis in macrophages and mice requires the glyoxylate shunt enzyme isocitrate lyase. Nature 406:735–738. doi:10.1038/35021074. 10963599

[26] Muñoz-Elías EJ, Upton AM, Cherian J, McKinney JD 2006 Role of the methylcitrate cycle in Mycobacterium tuberculosis metabolism, intracellular growth, and virulence. Mol Microbiol 60:1109–1122. doi:10.1111/j.1365-2958.2006.05155.x. 16689789

[27] Marrero J, Rhee KY, Schnappinger D, Pethe K, Ehrt S 2010 Gluconeogenic carbon flow of tricarboxylic acid cycle intermediates is critical for Mycobacterium tuberculosis to establish and maintain infection. Proc Natl Acad Sci U S A 107:9819–9824. doi:10.1073/pnas.1000715107. 20439709 PMC2906907

[28] Trujillo C, Blumenthal A, Marrero J, Rhee KY, Schnappinger D, Ehrt S 2014 Triosephosphate isomerase is dispensable in vitro yet essential for Mycobacterium tuberculosis to establish infection. mBio 5:e00085. doi:10.1128/mBio.00085-14. 24757211 PMC3994511

[29] Kalscheuer R, Weinrick B, Veeraraghavan U, Besra GS, Jacobs WR 2010 Trehalose-recycling ABC transporter LpqY-SugA-SugB-SugC is essential for virulence of Mycobacterium tuberculosis. Proc Natl Acad Sci U S A 107:21761–21766. doi:10.1073/pnas.1014642108. 21118978 PMC3003129

[30] Marrero J, Trujillo C, Rhee KY, Ehrt S 2013 Glucose phosphorylation is required for Mycobacterium tuberculosis persistence in mice. PLoS Pathog 9:e1003116. doi:10.1371/journal.ppat.1003116. 23326232 PMC3542180

[31] Homolka S, Niemann S, Russell DG, Rohde KH 2010 Functional genetic diversity among Mycobacterium tuberculosis complex clinical isolates: delineation of conserved core and lineage-specific transcriptomes during intracellular survival. PLoS Pathog 6:e1000988. doi:10.1371/journal.ppat.1000988. 20628579 PMC2900310

[32] Sassetti CM, Rubin EJ 2003 Genetic requirements for mycobacterial survival during infection. Proc Natl Acad Sci U S A 100:12989–12994. doi:10.1073/pnas.2134250100. 14569030 PMC240732

[33] Schnappinger D, Ehrt S, Voskuil MI, Liu Y, Mangan JA, Monahan IM, Dolganov G, Efron B, Butcher PD, Nathan C, Schoolnik GK 2003 Transcriptional adaptation of Mycobacterium tuberculosis within macrophages: insights into the phagosomal environment. J Exp Med 198:693–704. doi:10.1084/jem.20030846. 12953091 PMC2194186

[34] Rohde KH, Veiga DFT, Caldwell S, Balázsi G, Russell DG 2012 Linking the transcriptional profiles and the physiological states of Mycobacterium tuberculosis during an extended intracellular infection. PLoS Pathog 8:e1002769. doi:10.1371/journal.ppat.1002769. 22737072 PMC3380936

[35] Rohde KH, Abramovitch RB, Russell DG 2007 Mycobacterium tuberculosis invasion of macrophages: linking bacterial gene expression to environmental cues. Cell Host Microbe 2:352–364. doi:10.1016/j.chom.2007.09.006. 18005756

[36] Ehrt S, Rhee K 2013 Mycobacterium tuberculosis metabolism and host interaction: mysteries and paradoxes. Curr Top Microbiol Immunol 374:163–188. doi:10.1007/82_2012_299. 23242856

[37] de Carvalho LPS, Fischer SM, Marrero J, Nathan C, Ehrt S, Rhee KY 2010 Metabolomics of Mycobacterium tuberculosis reveals compartmentalized co-catabolism of carbon substrates. Chem Biol 17:1122–1131. doi:10.1016/j.chembiol.2010.08.009. 21035735

[38] Bordbar A, Lewis NE, Schellenberger J, Palsson BØ, Jamshidi N 2010 Insight into human alveolar macrophage and M. tuberculosis interactions via metabolic reconstructions. Mol Syst Biol 6:422. doi:10.1038/msb.2010.68. 20959820 PMC2990636

[39] Theus SA, Cave MD, Eisenach KD 2004 Activated THP-1 cells: an attractive model for the assessment of intracellular growth rates of Mycobacterium tuberculosis isolates. Infect Immun 72:1169–1173. doi:10.1128/IAI.72.2.1169-1173.2004. 14742569 PMC321586

[40] Fuhrer T, Heer D, Begemann B, Zamboni N 2011 High-throughput, accurate mass metabolome profiling of cellular extracts by flow injection-time of flight mass spectrometry. Anal Chem 83:7074–7080. doi:10.1021/ac201267k. 21830798

[41] Cappiello A, Famiglini G, Palma P, Trufelli H 2010 Matrix effects in liquid chromatography-mass spectrometry. J Liq Chromatogr Relat Technol 33:1067–1081. doi:10.1080/10826076.2010.484314.

[42] Kanehisa M, Goto S 2000 KEGG: Kyoto Encyclopedia of Genes and Genomes. Nucleic Acids Res 28:27–30. doi:10.1093/nar/28.1.27. 10592173 PMC102409

[43] Layre E, Sweet L, Hong S, Madigan CA, Desjardins D, Young DC, Cheng TY, Annand JW, Kim K, Shamputa IC, McConnell MJ, Debono CA, Behar SM, Minnaard AJ, Murray M, Barry CE, Matsunaga I, Moody DB 2011 A comparative lipidomics platform for chemotaxonomic analysis of Mycobacterium tuberculosis. Chem Biol 18:1537–1549. doi:10.1016/j.chembiol.2011.10.013. 22195556 PMC3407843

[44] Zimmermann M, Kuehne A, Boshoff HI, Barry CE, Zamboni N, Sauer U 2015 Dynamic exometabolome analysis reveals active metabolic pathways in non-replicating mycobacteria. Environ Microbiol 17:4802–4815. doi:10.1111/1462-2920.13056. 26373870 PMC10500702

[45] Subramanian A, Tamayo P, Mootha VK, Mukherjee S, Ebert BL, Gillette MA, Paulovich A, Pomeroy SL, Golub TR, Lander ES, Mesirov JP 2005 Gene set enrichment analysis: a knowledge-based approach for interpreting genome-wide expression profiles. Proc Natl Acad Sci U S A 102:15545–15550. doi:10.1073/pnas.0506580102. 16199517 PMC1239896

[46] Wishart DS, Jewison T, Guo AC, Wilson M, Knox C, Liu Y, Djoumbou Y, Mandal R, Aziat F, Dong E, Bouatra S, Sinelnikov I, Arndt D, Xia J, Liu P, Yallou F, Bjorndahl T, Perez-Pineiro R, Eisner R, Allen F, Neveu V, Greiner R, Scalbert A 2013 HMDB 3.0—the Human Metabolome Database in 2013. Nucleic Acids Res 41:D801–D807. doi:10.1093/nar/gks1065. 23161693 PMC3531200

[47] Peyron P, Vaubourgeix J, Poquet Y, Levillain F, Botanch C, Bardou F, Daffé M, Emile J-F, Marchou B, Cardona P-J, de Chastellier C, Altare F 2008 Foamy macrophages from tuberculous patients’ granulomas constitute a nutrient-rich reservoir for M. tuberculosis persistence. PLoS Pathog 4:e1000204. doi:10.1371/journal.ppat.1000204. 19002241 PMC2575403

[48] Daniel J, Maamar H, Deb C, Sirakova TD, Kolattukudy PE 2011 Mycobacterium tuberculosis uses host triacylglycerol to accumulate lipid droplets and acquires a dormancy-like phenotype in lipid-loaded macrophages. PLoS Pathog 7:e1002093. doi:10.1371/journal.ppat.1002093. 21731490 PMC3121879

[49] Beatty WL, Rhoades ER, Ullrich HJ, Chatterjee D, Heuser JE, Russell DG 2000 Trafficking and release of mycobacterial lipids from infected macrophages. Traffic 1:235–247. doi:10.1034/j.1600-0854.2000.010306.x. 11208107

[50] van den Elzen P, Garg S, León L, Brigl M, Leadbetter EA, Gumperz JE, Dascher CC, Cheng T-Y, Sacks FM, Illarionov PA, Besra GS, Kent SC, Moody DB, Brenner MB 2005 Apolipoprotein-mediated pathways of lipid antigen presentation. Nature 437:906–910. doi:10.1038/nature04001. 16208376

[51] Podinovskaia M, Lee W, Caldwell S, Russell DG 2013 Infection of macrophages with Mycobacterium tuberculosis induces global modifications to phagosomal function. Cell Microbiol 15:843–859. doi:10.1111/cmi.12092. 23253353 PMC3620910

[52] Côtes K, Bakala N’goma JC, Dhouib R, Douchet I, Maurin D, Carrière F, Canaan S 2008 Lipolytic enzymes in Mycobacterium tuberculosis. Appl Microbiol Biotechnol 78:741–749. doi:10.1007/s00253-008-1397-2. 18309478

[53] Peirs P, Lefèvre P, Boarbi S, Wang X-M, Denis O, Braibant M, Pethe K, Locht C, Huygen K, Content J 2005 Mycobacterium tuberculosis with disruption in genes encoding the phosphate binding proteins PstS1 and PstS2 is deficient in phosphate uptake and demonstrates reduced in vivo virulence. Infect Immun 73:1898–1902. doi:10.1128/IAI.73.3.1898-1902.2005. 15731097 PMC1064925

[54] Rienksma RA, Suarez-Diez M, Mollenkopf H-J, Dolganov GM, Dorhoi A, Schoolnik GK, Martins Dos Santos VA, Kaufmann SH, Schaap PJ, Gengenbacher M 2015 Comprehensive insights into transcriptional adaptation of intracellular mycobacteria by microbe-enriched dual RNA sequencing. BMC Genomics 16:34. doi:10.1186/s12864-014-1197-2. 25649146 PMC4334782

[55] Galagan JE, Sisk P, Stolte C, Weiner B, Koehrsen M, Wymore F, Reddy TBK, Zucker JD, Engels R, Gellesch M, Hubble J, Jin H, Larson L, Mao M, Nitzberg M, White J, Zachariah ZK, Sherlock G, Ball CA, Schoolnik GK 2010 TB database 2010: overview and update. Tuberculosis 90:225–235. doi:10.1016/j.tube.2010.03.010. 20488753

[56] Schubert OT, Ludwig C, Kogadeeva M, Zimmermann M, Rosenberger G, Gengenbacher M, Gillet LC, Collins BC, Röst HL, Kaufmann SHE, Sauer U, Aebersold R 2015 Absolute proteome composition and dynamics during dormancy and resuscitation of Mycobacterium tuberculosis. Cell Host Microbe 18:96–108. doi:10.1016/j.chom.2015.06.001. 26094805

[57] VanderVen BC, Fahey RJ, Lee W, Liu Y, Abramovitch RB, Memmott C, Crowe AM, Eltis LD, Perola E, Deininger DD, Wang T, Locher CP, Russell DG 2015 Novel inhibitors of cholesterol degradation in Mycobacterium tuberculosis reveal how the bacterium’s metabolism is constrained by the intracellular environment. PLoS Pathog 11:e1004679. doi:10.1371/journal.ppat.1004679. 25675247 PMC4335503

[58] Upton AM, McKinney JD 2007 Role of the methylcitrate cycle in propionate metabolism and detoxification in Mycobacterium smegmatis. Microbiology 153:3973–3982. doi:10.1099/mic.0.2007/011726-0. 18048912

[59] Yang X, Nesbitt NM, Dubnau E, Smith I, Sampson NS 2009 Cholesterol metabolism increases the metabolic pool of propionate in Mycobacterium tuberculosis. Biochemistry 48:3819–3821. doi:10.1021/bi9005418. 19364125 PMC2771735

[60] King ZA, Lu J, Dräger A, Miller P, Federowicz S, Lerman JA, Ebrahim A, Palsson BO, Lewis NE 2016 BiGG Models: a platform for integrating, standardizing and sharing genome-scale models. Nucleic Acids Res 44:D515–D522. doi:10.1093/nar/gkv1049. 26476456 PMC4702785

[61] Fontán P, Aris V, Ghanny S, Soteropoulos P, Smith I 2008 Global transcriptional profile of Mycobacterium tuberculosis during THP-1 human macrophage infection. Infect Immun 76:717–725. doi:10.1128/IAI.00974-07. 18070897 PMC2223452

[62] Pethe K, Sequeira PC, Agarwalla S, Rhee K, Kuhen K, Phong WY, Patel V, Beer D, Walker JR, Duraiswamy J, Jiricek J, Keller TH, Chatterjee A, Tan MP, Ujjini M, Rao SPS, Camacho L, Bifani P, Mak PA, Ma I, Barnes SW, Chen Z, Plouffe D, Thayalan P, Ng SH, Au M, Lee BH, Tan BH, Ravindran S, Nanjundappa M, Lin X, Goh A, Lakshminarayana SB, Shoen C, Cynamon M, Kreiswirth B, Dartois V, Peters EC, Glynne R, Brenner S, Dick T 2010 A chemical genetic screen in Mycobacterium tuberculosis identifies carbon-source-dependent growth inhibitors devoid of in vivo efficacy. Nat Commun 1:57. doi:10.1038/ncomms1060. 20975714 PMC3220188

[63] Shlomi T, Cabili MN, Herrgård MJ, Palsson BØ, Ruppin E 2008 Network-based prediction of human tissue-specific metabolism. Nat Biotechnol 26:1003–1010. doi:10.1038/nbt.1487. 18711341

[64] Russell DG, Barry CE, Flynn JL 2010 Tuberculosis: what we don’t know can, and does, hurt us. Science 328:852–856. doi:10.1126/science.1184784. 20466922 PMC2872107

[65] Griffin JE, Gawronski JD, Dejesus MA, Ioerger TR, Akerley BJ, Sassetti CM 2011 High-resolution phenotypic profiling defines genes essential for mycobacterial growth and cholesterol catabolism. PLoS Pathog 7:e1002251. doi:10.1371/journal.ppat.1002251. 21980284 PMC3182942

[66] Dietrich G, Schaible UE, Diehl KD, Mollenkopf H, Wiek S, Hess J, Hagens K, Kaufmann SH, Knapp B 2000 Isolation of RNA from mycobacteria grown under in vitro and in vivo conditions. FEMS Microbiol Lett 186:177–180. doi:10.1111/j.1574-6968.2000.tb09100.x. 10802167

[67] Illumina 2014 TruSeq RNA sample preparation v2 guide. Illumina, San Diego, CA.

[68] Bolger AM, Lohse M, Usadel B 2014 Trimmomatic: a flexible trimmer for Illumina sequence data. Bioinformatics 30:2114–2120. doi:10.1093/bioinformatics/btu170. 24695404 PMC4103590

[69] Anders S, Huber W 2010 Differential expression analysis for sequence count data. Genome Biol 11:R106. doi:10.1186/gb-2010-11-10-r106. 20979621 PMC3218662

